# Computed Tomography Study of the Retrosigmoid Craniotomy Keyhole Approach Using Surface Landmarks

**DOI:** 10.1155/2023/5407912

**Published:** 2023-03-01

**Authors:** Wei Hu, Jiang Zhou, Zheng-Min Liu, Ye Li, Qing-Feng Cai, Xiao-Ming Hu, Yan-Bing Yu

**Affiliations:** ^1^Department of Neurosurgery, Taizhou Hospital of Zhejiang Province Affiliated to Wenzhou Medical University, Taizhou 317000, China; ^2^Department of Neurosurgery, Enze Medical Center (Group) Enze Hospital, Taizhou 318050, China; ^3^Department of Neurosurgery, China-Japan Friendship Hospital, Beijing 100029, China

## Abstract

**Background:**

Due to a lack of accessibility and individual differences in surgical procedures, many previous studies on keyholes are not practical.

**Objective:**

To study the surface landmarks for optimal keyhole placement in the retrosigmoid approach.

**Methods:**

The three-dimensional (3D) skull images of 79 patients were reconstructed using workstations, with a total of 149 hemiskull base 3D images then analyzed. Skull-surface landmarks were marked, the lateral-skull surface was observed, and the positional relationships between the asterion and the extension line of the posterior margin of the mastoid process were measured. The position of the superior curvature of the sigmoid sinus groove was located before it was projected onto the lateral surface of the skull and defined as the keypoint. The positional relationship between the keypoint and the skull-surface landmarks was observed in an established coordinate system using spatial proportion relationships.

**Results:**

The asterion was located around the extension line of the posterior margin of the mastoid process, and the vertical distance from the extension line was <15 mm. It was found that 93.29% (139/149) of the keypoints were located in a 7 mm radius circle, with the center at (−0.41, −3.01) in the coordinate system in the 3D computed tomography images.

**Conclusion:**

When using this method, the spatial proportion relationship of the anatomical marks can accurately locate keyholes, therefore providing technical support when employing the retrosigmoid approach.

## 1. Introduction

The retrosigmoid approach is commonly utilized for the surgical pathology of the cerebellopontine angle (CPA) and the lateral cerebellar hemisphere. The craniotomy for this approach must expose the posterior edge of the superior curvature of the sigmoid sinus, since the dura mater should be opened as close as possible to this landmark to provide adequate exposure of the lateral posterior fossa, thus reducing the need for brain retraction. The initial burr hole for retrosigmoid craniotomy is known as the keyhole and should be placed posterior to the superior curvature of the sigmoid sinus and inferior to the transverse sinus. Correct placement requires the surgeon to correctly identify the location of the cranial surface projection of the transverse-sigmoid junction. Most identification methods use the asterion as the reference point for this location [1–6]. However, Bozbuga et al. [7] and da Silva et al. [8] present key studies (anatomical, radiological, and clinical) reporting that the asterion is not a reliable landmark for burr hole placement in the retrosigmoid approach. Indeed, an increasing number of studies have demonstrated that there is considerable anatomic variation in the relationship between the asterion and the transverse-sigmoid junction, and incorrect venous sinus localization may result in sinus injury or insufficient surgical exposure [2, 9–11]. While other skull-surface landmarks are also used to locate the transverse-sigmoid junction, they have anatomic variations, and some are too far from the surgical incision to be practical for surgical use [12]. Three-dimensional (3D) computed tomography (CT) image-guided surgery allows for correct craniotomy placement but is not applicable in hospitals lacking the necessary equipment or for patients with contrast hypersensitivity, nor is it practical in emergency surgery [13]. However, an anatomical study of surface landmarks and their relationship with the transverse-sigmoid junction can provide relevant information regarding accurate retrosigmoid craniotomy placement without image guidance.

Previous studies have focused on the distance between surface landmarks and keyholes, which is shown to be greatly affected by the differences in 3D morphology among different individuals. It was hypothesized that the relationship between skull-surface landmarks and the keyhole follows spatial proportionality. To avoid this defect and verify the hypothesis, this study aims to examine the relationship between skull-surface landmarks and the keyhole using line segment proportions rather than length in 3D CT image reconstruction.

## 2. Materials and Methods

This study involved collecting and studying existing data, documents, or records, all of which were publicly available. The Institutional Review Board of Enze Medical Center (Group) and Enze Hospital reviewed this study and determined that it is exempt from the full ethics committee review, with informed consent thus waived.

### 2.1. Criteria of Three-Dimensional Skull Images

This study was an estimation survey of the overall rate in counting data, aiming for a positive rate with a keypoint within a specific-diameter circle. The difference between the sample rate (a) and the overall rate (P) was expected to be no more than 10%. Based on the sample rate of a small-scale presurvey, *P*=80% and *a* = 0.05, while the sample size = (Ua/*δ*)/*P*(1 − *P*), where *δ* = 0.10, *P*=0.88, *a* = 0.05, and Ua = 1.96 (*U α* is the *U* value corresponding to the significance level, and *δ* is error).

The expected sample size was finally calculated as 123 cases. Finally, for this study, 79 patients who underwent thin-section CT of the head in Enze Medical Center (Group), Enze Hospital, were randomly selected for an anatomical study. All the patients were Chinese nationals, including 97 male and 52 female patients. The minimum age was nine years old, and the maximum was 78, with 74 cases involving the left side of the skull and 75 cases involving the right. Imaging data were reconstructed using workstation (GE ADW4.6) 3D CT software (General Electric Company, USA) to create 3D skull images.

Images containing any unclear surface markers were excluded. A total of 149 hemiskull base 3D images from 79 patients were adequate for analysis (clear visualization of the entire mastoid process, the asterion, the digastric groove apex, the external auditory canal, the transverse sinus sulcus, and the sigmoid sinus sulcus).

### 2.2. The Apex of the Superior Curvature of the Sigmoid Sinus Groove

The relevant literature mostly discusses using the transverse-sinus-sigmoid junction or the superior curvature of the sigmoid sinus to set the keyhole, and only a few studies provide a clear explanation of this process [14].

**Figure 1:**
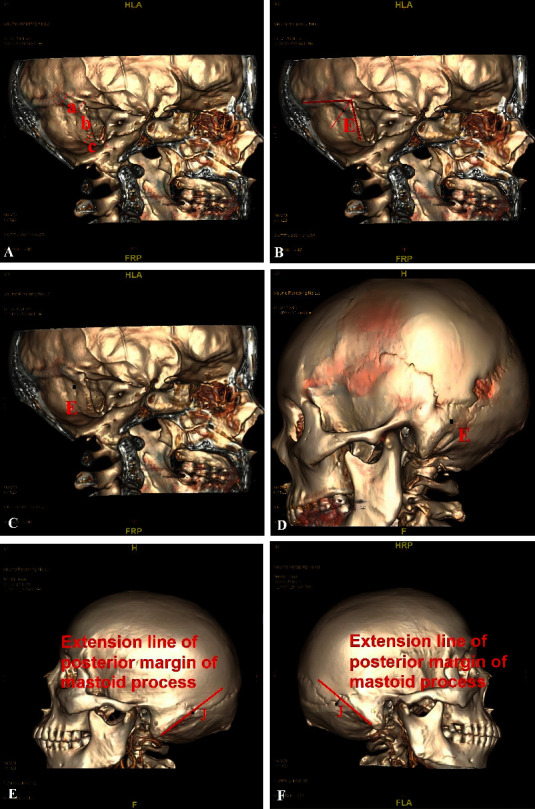
Marking and measurement. (A) The sigmoid sinus was divided into three parts: (a) superior curvature, (b) vertical segment, and (c) inferior curvature. (B) The E point at the inner edge of the superior curvature of the sigmoid sinus groove along the angular bisector of the intersection of the lines passing through the central axis of the transverse sinus and the vertical segment of the sigmoid sinus was defined as the keypoint on the medial surface of the skull. (C) The apex of the superior curvature of the sigmoid sinus groove was found by the method described in Figure 1. (D) The apex of the superior curvature of the sigmoid sinus groove was vertically cut to form a hole with a diameter of 1 mm using a workstation. The hole's center point, E, was the keypoint on the outer surface of the skull. (E, F) The extension line of the posterior margin of the mastoid process was drawn, and the vertical distance between it and the asterion (point J) was measured.

In this study, the apex of the superior curvature of the sigmoid sinus groove on the 3D CT skull reconstruction images was used to determine the location of the keypoint on the medial surface of the skull. The sigmoid sinus was divided into three parts: superior curvature, vertical segment, and inferior curvature. The superior curvature is adjacent to the lower boundary of the outer edge of the tentorium. It begins where the transverse sinus ends, at the point where the sinus starts to curve inferiorly, and it ends where the curvature stops, which is where the vertical segment begins. Point E was defined as the keypoint on the medial surface of the skull. It was at the inner edge of the superior curvature of the sigmoid sinus along the angular bisector of the intersection of the lines passing through the central axis of the transverse sinus and the vertical segment of the sigmoid sinus (Figures 1(a) and 1(b)). Point E was the projection of this point on the lateral surface of the skull (Figures 1(c) and 1(d)), while the hole that was drilled with the keypoint as its center was the keyhole.

### 2.3. Lateral Skull Surface

The positional relationship between the asterion and the skull-surface landmarks was quantified. First, the sight perpendicular to the external auditory canal of the 3D image and the line of the posterior margin of the mastoid process were located and extended to the cranial side. A vertical line was then drawn from the asterion to the extension line, and the vertical distance between them was measured (Figures 1(e) and 1(f)).

**Figure 2:**
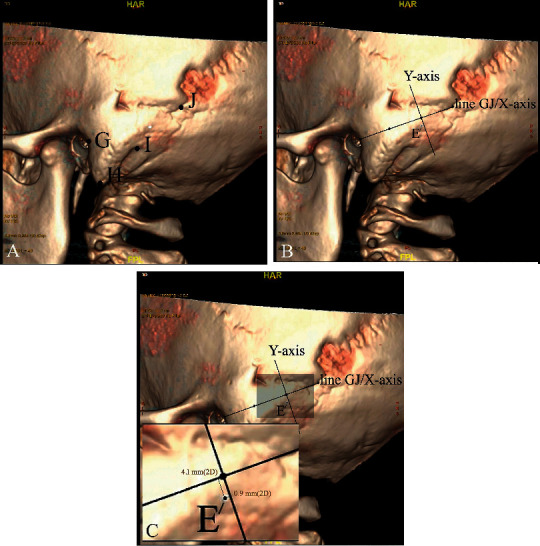
Lateral-skull surface and coordinate of point E; (a) on the lateral surface of the 3D image, with the line of sight perpendicular to the plane near the keypoint, the following points were marked: the midpoint of the posterior edge of the external auditory canal (point G), the mastoid process apex (point H), the digastric groove apex (point I), and the asterion (point J). All these landmarks were close to the keypoint, meaning they were easy to palpate intraoperatively. (b) Line GJ was the *x*-axis; a point below one-third of the GJ-line segment was the origin; and a perpendicular line running from the origin was the *y*-axis. The coordinate system was then established. (c) The coordinates of point E were determined via the coordinate system using a workstation.

On the lateral surface of the 3D image, the following points were marked: the midpoint of the posterior edge of the external auditory canal (point G), the mastoid process apex (point H), the digastric groove apex (point I), and the asterion (point J) (Figure 2(a)).

A coordinate system was established on the lateral surface of the 3D image using the GJ line as the *x*-axis, a point below one-third of the GJ-line segment as the origin, and a perpendicular line from the origin as the *y*-axis. The coordinates of point E were determined in the coordinate system using the workstation (Figures 2(b) and 2(c)).

### 2.4. Statistics

The statistical analysis was performed using SPSS 16.0 software (SPSS, Inc., Chicago, Illinois, USA). For the continuous variables, the data conforming to the normal distribution were expressed in the form of mean ± standard deviation (SD), with the Kolmogorov–Smirnov used to test the normal distribution.

## 3. Results

### 3.1. Comparison of Measurement Results of Different Genders and Sides

The *X* and *Y* values of patients of different genders, ages, and sides were measured, and no significant difference was found (Table 1).

### 3.2. Asterion

The mean distance from the asterion to the extension line of the posterior margin of the mastoid process was 6.74 mm (range = 0.50–15.30 mm, SD = 3.69 mm). Regarding the relationship between the asterion and the extension line, the asterion was on the ventral side of the extension line in 65.89% of cases and on the dorsal side in 34.11% of cases.

It was observed that the asterion was clearly located close to the extension line, with the vertical distance between them ranging from 0–15 mm in most cases (97.98%; 146/149). The vertical distance between the asterion and the extension line was generally consistent with the normal distribution (skewness coefficient = 0.504; kurtosis = −0.206).

### 3.3. Keypoint Coordinates

**Figure 3:**
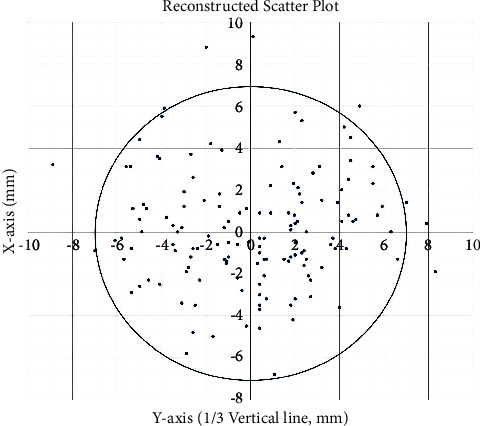
Keypoint coordinates show that 89.77% (139/149) of the keypoints were located within a 7 mm radius circle, with the center at (−0.41, −3.01) in the coordinate system.

The coordinates of the projection of the keypoint on the lateral surface of the skull (point E) are shown in the scatter plot presented in Figure 3. The mean *x* coordinate was −0.41 mm, and the mean *y* coordinate was −3.01 mm. The point coordinates (−0.41, −3.01) were reset as the origin, and the scatterplot was reconstructed. The result revealed that 93.29% (139/149) of the keypoints were located within a 7-mm-radius circle, with the center at (−0.41, −3.01) in the coordinate system in the 3D CT images (Figure 3).

The mean distance from the keypoint to the origin was 3.86 mm (range = 0.38–9.45 mm, SD = 2.00 mm). The distance was mostly consistent with the normal distribution (skewness coefficient = 0.486; kurtosis = −0.281).

## 4. Discussion

### 4.1. Background

The retrosigmoid approach is commonly used for the surgical treatment of various posterior fossa pathologies. The surgical field can be adjusted as needed to expose the anatomical structures and lesions located below the tentorium cerebelli in the CPA and lateral cerebellum. Due to limited space in the posterior fossa, the craniotomy in this procedure should expose the transition between the transverse and sigmoid sinuses to fully reveal the lesion, acquire adequate visualization and access, and reduce retraction injury to the cerebellum. Therefore, dural exposure should include the posterior edge of the superior curvature of the sigmoid sinus superiorly and the posterior edge of the vertical segment anteriorly. To achieve this, the bone flap needs to be as close to the transverse and sigmoid sinuses as possible. Identifying the skull-surface projections of the superior curvature and vertical segment of the sigmoid sinus prior to craniotomy enables accurate placement of the critical first burr hole, i.e., the keyhole, to safely expose the transverse-sigmoid junction without causing venous sinus injury or bleeding.

The asterion is defined as the intersection of the occipitomastoid, parietomastoid, and lambdoid sutures [15]. While it has been widely considered to overlie the superior curvature of the sigmoid sinus, more recent studies have noted significant anatomical variations in this positional relationship [2, 9–11]. The spatial relationship between the asterion and the superior curvature of the sigmoid sinus is not constant from one individual to another and cannot be relied on to locate the keyhole for a retrosigmoid craniotomy accurately. Keyhole drilling using the asterion or other surface landmarks as reference points may result in poor exposure or surgical failure or may require increased skull destruction or a prolonged operation time. Other potentially severe consequences include life-threatening venous sinus injury, air embolism, venous infarction, and massive hemorrhaging.

### 4.2. Locating the Keypoint

Here, the spatial proportion relationship of the anatomical marks on the patient's skull surface was used to locate keypoints, with 3D hemilateral skull base CT images used to locate the keypoint required for performing a retrosigmoid craniotomy, i.e., the apex of the superior curvature of the sigmoid sinus [2]. After establishing reference lines and a coordinate system, the relationships of the keypoint, transverse, and sigmoid sinuses to skull-surface landmarks could be measured and analyzed in 79 patients, and it was found that the keypoint was located close to a point below one-third of the GJ-line segment. Knowledge of these relationships should allow for accurate localization of the keypoint and venous sinuses without image guidance and should result in safer, faster, and less invasive retrosigmoid craniotomies.

Matsuo et al. [16] proposed using the relationship between the horizontal part of the sigmoid sinus and the line through the digastric point and posterior edge of the condyle to locate the optimum area for the burr hole placement, which has a number of similarities to the method used in the current study. This method was found to be intuitive, accurate, and easy to perform. The asterion was located close to the extension line in the images analyzed, specifically within 15 mm of the extension line of the posterior margin of the mastoid process in 96.59% of the images. The keypoint was usually located within a 7 mm-radius circle centered on a point around 3 mm below one-third of the line segment between the midpoint of the posterior edge of the external auditory canal and the asterion (89.77%). The spatial relationship between the anatomic structures and the surface points demonstrated here can provide an anatomical basis for craniotomy placement in emergency cases or when 3D CT imaging or surgical navigation are unavailable.

### 4.3. Generalizability

When planning a retrosigmoid craniotomy during surgery, relevant bony landmarks (Figure 2(a)) can be easily palpated intraoperatively, thus identifying the keypoint. The location of the asterion can be estimated by palpating a bony depression 0–15 mm from the extension line of the posterior margin of the mastoid process. The midpoint of the posterior edge of the external auditory canal can be palpated directly. A burr hole with a center point 3 mm below one-third of the GJ line segment, as described above (Figure 2), is made with the drill. Taking point E of this study as the center of the circle (Figure 2), a craniotomy drill (with a diameter of 12–15 mm) can be used to drill holes, and a bone hole with a diameter of around 14 mm can be obtained. The apex of the superior sigmoid sinus can be exposed in the bone hole of more than 90% of patients.

Therefore, based on this study, the deviation in individual cases can be corrected by obtaining bone holes with a craniotomy drill. If the sinus cannot be seen through the bone hole, a drill or a rongeur can be used to remove more bone anteriorly until the edge of the superior curvature of the sigmoid sinus is exposed. Once the keypoint is identified, the craniotomy can be tailored according to specific needs. For example, craniotomy for microvascular decompression (MVD) of the trigeminal nerve should be performed at the keyhole, facial-nerve MVD should be performed approximately 5 mm below the keyhole, and glossopharyngeal-nerve MVD should be performed 5–10 mm below the keyhole. Craniotomy involving the bone flap and the keyhole is precisely designed to treat various cranial nerve diseases, which further reduces the risk of postoperative cerebrospinal fluid leakage in addition to decreasing the craniotomy-related trauma and the risk of bleeding. However, keyhole treatment is not recommended for patients with recurrence, trigeminal neuralgia complicated by glossopharyngeal neuralgia, or a small posterior fossa volume.

Despite the considerable anatomic variation among individuals, the superior curvature of the sigmoid sinus was accurately identified in over 93% of patients using a line connecting the midpoint of the posterior edge of the external auditory canal to the asterion as a reference. While modern 3D CT, CT venography, and image-fusion technology will become more common in the future, it is still necessary to combine advanced imaging techniques with basic morphological studies to clarify important surgically relevant anatomical relationships. In the new method in this study, the spatial proportion relationship of the anatomical marks on the patient's skull surface can be used to locate keypoints, which was validated via postsurgery 3D CT images (Supplementary Figures 1–3). Similar studies on the protection of the superior sagittal sinus during bicoronal craniotomies and the transverse sinus during craniotomies for other posterior fossa approaches are required. The next step in this research will be to use the same method to measure dry skulls to further verify the role of surface landmarks in locating the retrosigmoid keyhole.

A positioning method and a novel research idea were both presented in this paper. Using spatial proportion instead of distance may better reflect the anatomical reality of individual differences.

## 5. Limitations

The main purpose of this study was to clarify intrinsic, surgically relevant anatomical relationships between the keyhole and the skull-surface landmarks from a stereoscopic perspective. While over 93.29% of the sample population met expectations, just fewer than 6% of the samples still involved clear deviations (Figure 3). In addition, given that a 3D CT image is not an actual human skull, the next step will be to use human skulls for further verification.

## 6. Conclusion

The spatial proportion relationship of the anatomical marks on a patient's skull surface can be used to locate keypoints. This method can accurately identify keyholes in approximately 90% of patients. While keypoints can easily be located using 3D CT or CT venography preoperatively, it is still necessary to clarify important surgically relevant anatomical relationships and to combine advanced imaging techniques with basic morphological studies. This study introduced one method that will help achieve the main goal.

## Figures and Tables

**Table 1:** Comparison of measurement results of different genders and sides.

Index	*N*	*X*	*Y*
<50 y	67	0.10 ± 3.59	−2.74 ± 2.74
≥50 y	82	−0.82 ± 3.28	−3.23 ± 2.61
*P* value		0.106^*∗*^	0.265^*∗*^
Male	97	−0.46 ± 3.47	−2.78 ± 2.82
Female	52	−0.30 ± 3.43	−3.43 ± 2.33
*P* value		0.793^*∗*^	0.156^*∗*^
Left	97	−0.19 ± 3.57	−2.93 ± 2.60
Right	52	−0.81 ± 3.19	−3.15 ± 2.81
*P* value		0.290^*∗*^	0.636^*∗*^

^*∗*^ *T*-test was used to compare the measurement results.

## Data Availability

All data generated or analyzed during this study are included in this article. Further enquiries can be directed to the corresponding author.

## Supplementary Materials

Supplementary Materials Supplementary Figure 1: the yellow line is the line midpoint of the posterior edge of the external auditory canal to the asterion, the pentacle is the located keypoint, and the yellow circle is the bone window designed for keyhole surgery. Supplementary Figure 2: after locating the keypoints according to the author's method, keyhole surgery for hemifacial spasm can be performed. As shown, it was an intraoperative bone window. Supplementary Figure 3: the bone window is shown in a 3D reconstruction of the skull following a hemifacial spasm.
