# Supplementary figures and images for: Computed Tomography Study of the Retrosigmoid Craniotomy Keyhole Approach Using Surface Landmarks
Source: Int J Clin Pract. 2023 Mar 1;2023:5407912. doi: 10.1155/2023/5407912 (PMC9995208; doi:10.1155/2023/5407912)

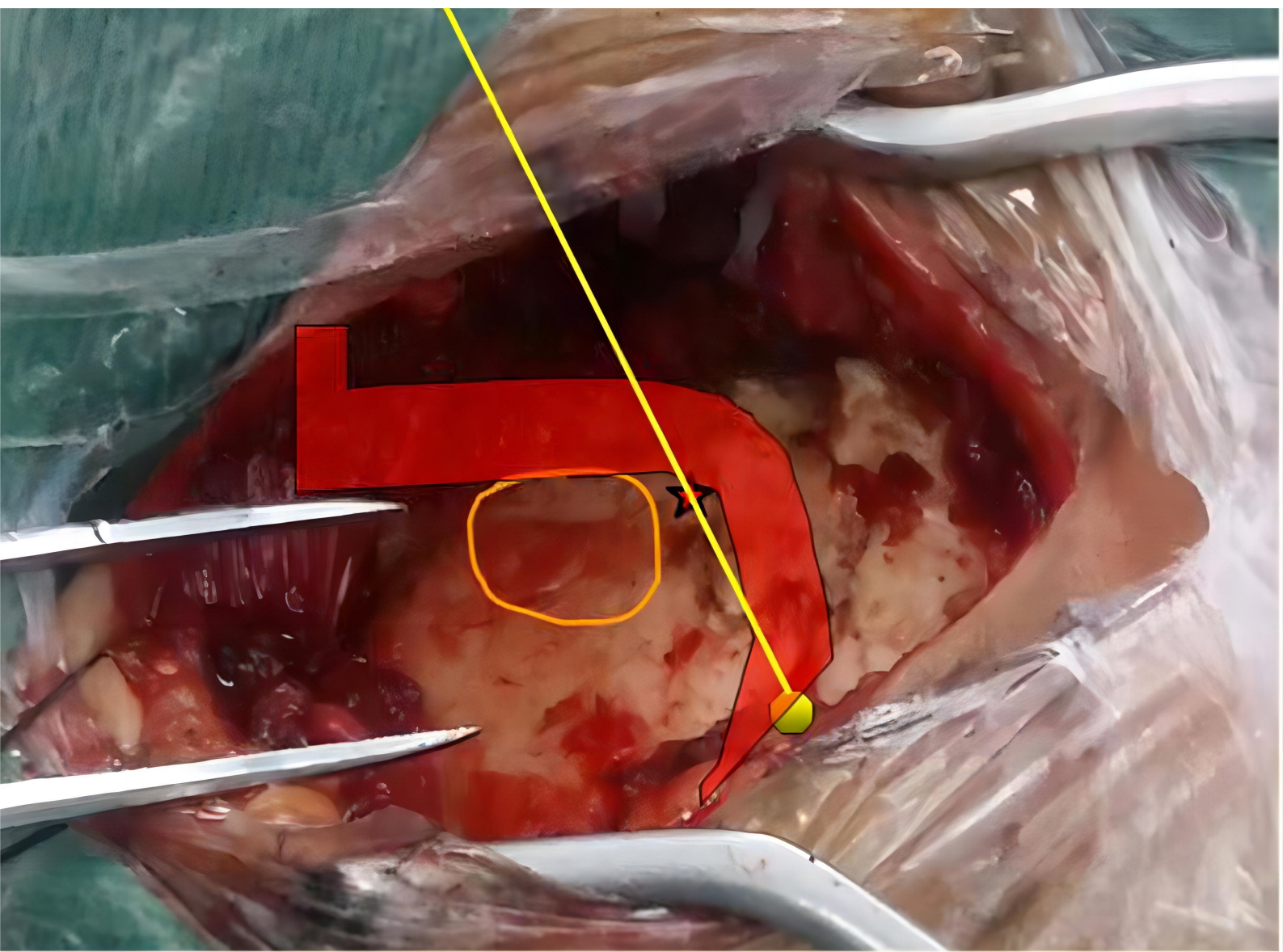

Supplement: Supplementary Materials — Supplementary Figure 1: the yellow line is the line midpoint of the posterior edge of the external auditory canal to the asterion, the pentacle is the located keypoint, and the yellow circle is the bone window designed for keyhole surgery. Supplementary Figure 2: after locating the keypoints according to the author's method, keyhole surgery for hemifacial spasm can be performed. As shown, it was an intraoperative bone window. Supplementary Figure 3: the bone window is shown in a 3D reconstruction of the skull following a hemifacial spasm. [file 5407912.f1.zip › Supplement Figure 1.pdf]

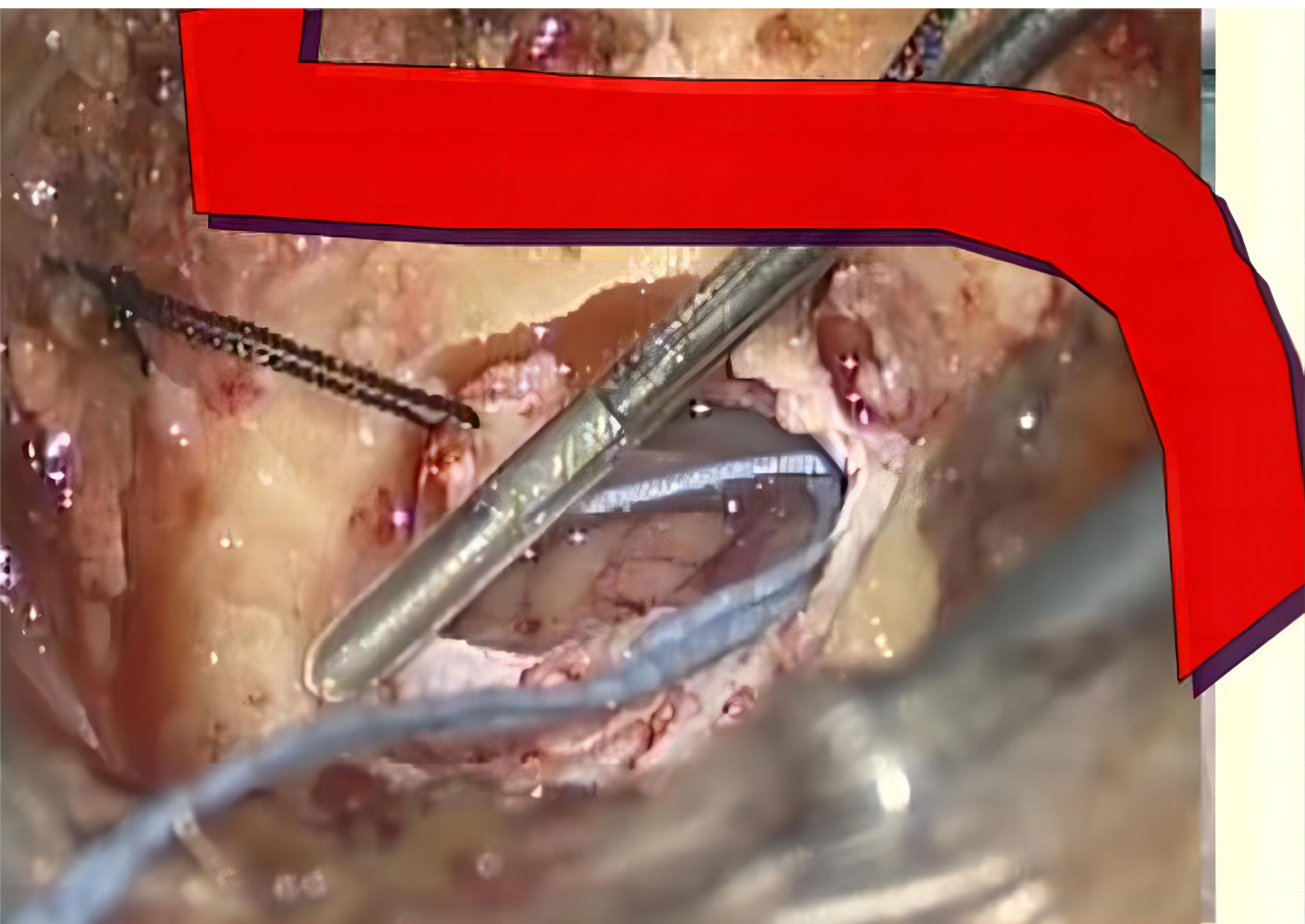

Supplement: Supplementary Materials — Supplementary Figure 1: the yellow line is the line midpoint of the posterior edge of the external auditory canal to the asterion, the pentacle is the located keypoint, and the yellow circle is the bone window designed for keyhole surgery. Supplementary Figure 2: after locating the keypoints according to the author's method, keyhole surgery for hemifacial spasm can be performed. As shown, it was an intraoperative bone window. Supplementary Figure 3: the bone window is shown in a 3D reconstruction of the skull following a hemifacial spasm. [file 5407912.f1.zip › Supplement Figure 2 .pdf]

Se:3  
Volume Rendering No cut

Non GE image  
DFOV 15.7 cm  
UB No Filter

P  
L  
S

P  
R  
H

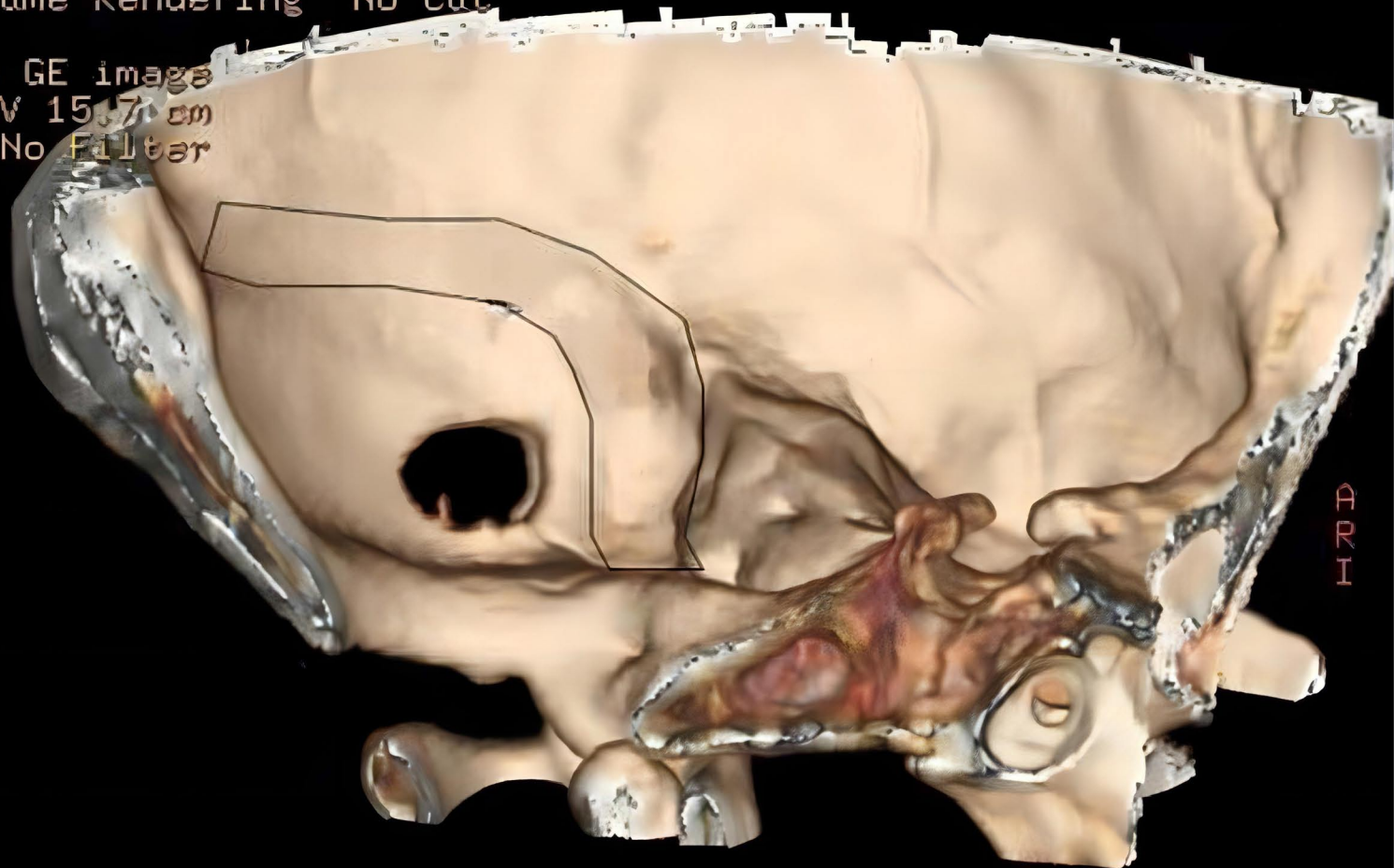

Supplement: Supplementary Materials — Supplementary Figure 1: the yellow line is the line midpoint of the posterior edge of the external auditory canal to the asterion, the pentacle is the located keypoint, and the yellow circle is the bone window designed for keyhole surgery. Supplementary Figure 2: after locating the keypoints according to the author's method, keyhole surgery for hemifacial spasm can be performed. As shown, it was an intraoperative bone window. Supplementary Figure 3: the bone window is shown in a 3D reconstruction of the skull following a hemifacial spasm. [file 5407912.f1.zip › Supplement Figure 3 .pdf]
